# Evolution of the digital operating room: the place of video technology in surgery

**DOI:** 10.1007/s00423-023-02830-7

**Published:** 2023-02-20

**Authors:** Samy Cheikh Youssef, Kaled Haram, Jonathan Noël, Vipul Patel, James Porter, Prokar Dasgupta, Nadine Hachach-Haram

**Affiliations:** 1 St. Georges University of London, London, UK; 2 Westminster School, London, UK; 3 Guy’s and St. Thomas’ NHS Foundation Trust, Urology Centre, King’s Health Partners, London, UK; 4 Adventhealth Global Robotics Institute, 400 Celebration Place, Celebration, FL USA; 5 Department of Urology, Swedish Urology Group, Seattle, WA USA; 6 Department of Plastic Surgery, Guy’s and St. Thomas’ NHS Foundation Trust, King’s Health Partners, London, UK

**Keywords:** Artificial intelligence, Robotic surgery, Surgical innovation, Surgical video, Video labelling

## Abstract

**Purpose:**

The aim of this review was to collate current evidence wherein digitalisation, through the incorporation of video technology and artificial intelligence (AI), is being applied to the practice of surgery. Applications are vast, and the literature investigating the utility of surgical video and its synergy with AI has steadily increased over the last 2 decades. This type of technology is widespread in other industries, such as autonomy in transportation and manufacturing.

**Methods:**

Articles were identified primarily using the PubMed and MEDLINE databases. The MeSH terms used were “surgical education”, “surgical video”, “video labelling”, “surgery”, “surgical workflow”, “telementoring”, “telemedicine”, “machine learning”, “deep learning” and “operating room”. Given the breadth of the subject and the scarcity of high-level data in certain areas, a narrative synthesis was selected over a meta-analysis or systematic review to allow for a focussed discussion of the topic.

**Results:**

Three main themes were identified and analysed throughout this review, (1) the multifaceted utility of surgical video recording, (2) teleconferencing/telemedicine and (3) artificial intelligence in the operating room.

**Conclusions:**

Evidence suggests the routine collection of intraoperative data will be beneficial in the advancement of surgery, by driving standardised, evidence-based surgical care and personalised training of future surgeons. However, many barriers stand in the way of widespread implementation, necessitating close collaboration between surgeons, data scientists, medicolegal personnel and hospital policy makers.

## Introduction

Technological advancements and applications of digitalisation to date reveal opportunities for surgical innovation. A consensus on the definition of digital surgery was reached in 2022 by Lam et al., defining *digital surgery* as “the use of technology for the enhancement of preoperative planning, surgical performance, therapeutic support, or training, to improve outcomes and reduce harm” [1].

Surgical adverse events remain a significant cause of mortality and morbidity in patients worldwide, with research suggesting that over 50% are preventable [2]. Over half of all surgical errors are due to underdeveloped performance pre-, intra- and post-operatively. Factors such as suboptimal planning, communication and poor execution were identified [3]. World Health Organisation (WHO) evidence states that robust and systematic approaches to patient safety can mitigate medical errors by 50–70.2% [4].

A significant challenge for the surgical community is the backlog of patients after the surges in the COVID-19 pandemic. In England and Wales, resource diversion resulted in a 33.6% reduction in surgical activity in 2020 [5]. In the USA, there was a greater reduction with mass cancellation of elective surgical procedures at 48.0% between 2019 and 2020 [6]. In the field of education, a review of surgical logbook data from 2019 to 2020 revealed a 50% reduction in operations with trainees as the primary operating surgeon leading to a reduction in operative experience [7]. However, incorporating digital solutions was prompt, with many institutions resorting to online platforms for knowledge learning [8].

In recent years, the utility of technology in the surgical field has become increasingly evident. The COVID-19 pandemic made evident the value of online learning in the form of video, telementoring and teleconferences, which are likely to become more prevalent in the coming years with the rise of blended learning, where online resources complement traditional training methods [9]. There has been an increased interest in augmented/virtual reality [10], surgical data science and AI analysing operative metrics to improve surgical performance, training and ultimately patient outcomes. Underlying these applications is the prerequisite for big data, which in surgery is achieved through the routine collection and utilisation of operative details [10]. Nonetheless, many ethical and logistic issues remain obstacles to the uptake of these technologies by health institutions.

**Fig. 1:**
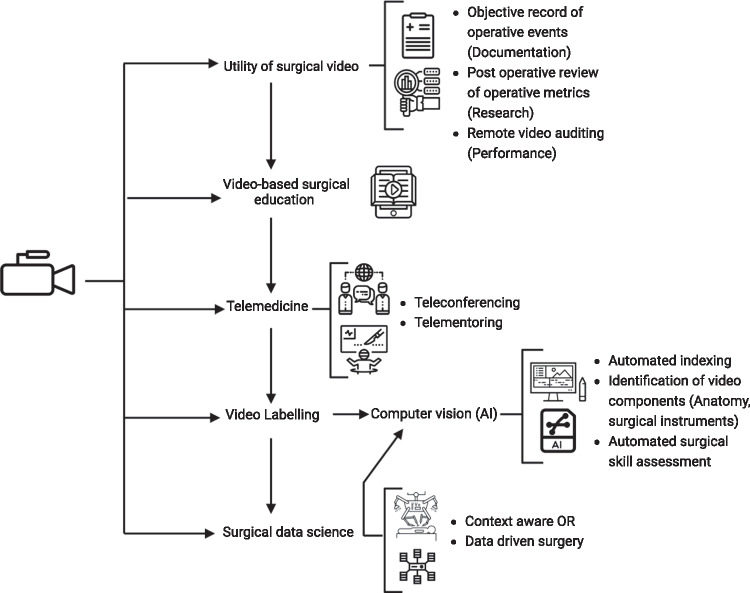
The place of video technology in surgery, study outline

This review will focus on how the stance of video technology and AI can augment current surgical practice and overcome these contemporary challenges; we discuss systematically the use of video in its basic form to its application as a source of data in AI (Fig. 1).

## Methods

Articles were identified primarily using the PubMed and MEDLINE databases. The MeSH terms used were “surgical education”, “surgical video”, “video labelling”, “surgery”, “surgical workflow”, “telementoring”, “telemedicine”, “machine learning”, “deep learning” and “operating room”. Reference lists were also explored for additional articles. Articles were selected for inclusion based on the relevance and appropriateness of its content as deemed by the expertise and knowledge of the authors. Given the breadth of the subject and the scarcity of high-level data in certain areas, a narrative synthesis was selected over a meta-analysis or systematic review to allow for a focussed discussion of the topic.

### Utility of surgical video

Surgical video offers an abundance of data, capturing sources of variability which can be analysed for personalised training interventions and systematic quality improvement. Such applications are discussed in this section.

Surgical video is an objective record of intraoperative events, enabling review and investigation of surgical performance and how intraoperative events correlate to patient outcomes. Birkmeyer et al. demonstrated that a surgeon’s skill, determined by peer review rating of video-recorded cases, has significant associations with operative time, length of stay and mortality for gastric bypass surgery [11]. A study in 2020 of 17 practicing surgeons confirmed this finding; over 25% of the complication rates were due to variations in technical skill scores graded in the surgical video, which varied greatly amongst practicing surgeons (2.8–4.6/5). Higher technical skill scores for the surgery under study (colectomy) were associated with lower rates of complications, unplanned reoperation and subsequently improved postoperative outcomes [12]. Conversely, in laparoscopic sleeve gastrectomy, surgical skill did not have a significant impact on overall complication rates, despite a wide variation in surgical skill scores (2.7–4.6/5). This was thought to be due to a shorter learning curve [13]. Through video analysis, differences in surgical technique between the top-scoring surgeons and lowest-scoring surgeons can be detected [13].

This can enhance surgical coaching through personalised training. The identification of optimal operative technique of the best performing surgeons and technical factors associated with operative complications presents an opportunity to bolster training.

Recorded video repositories provide an objective record for factors relating to a patient’s postoperative course, eliminating subjective elements to surgery. A 2011 study examined whether operative notes accurately represent laparoscopic cholecystectomy, when compared to video recordings; the results showed significant omissions of procedural steps in numerous operative reports [14]. Novel methods of automation can reduce such variation in operative documentation, using checklists based on business industry models, to prevent omission of important steps and enhance documentation [15].

Surgical video offers fine detail which can be analysed. Microanalysis of a video dataset identified human factors found to impact the surgical team’s performance and situational awareness [16]. Linguistic factors were identified, such as the use of open and closed questions and their effects on subsequent behaviour documented [16]. Beyond oral instruction, extrinsic sounds such as music and equipment alarms are also associated with technical error. Ayas et al. showed in gastric bypass surgery that machine alarms, which were one of the most common distractions, were associated with clinically relevant technical errors [17].

The awareness by surgical staff of video recording in the surgical setting may also be inherently advantageous. A study in 2018 showed there was a significant decrease in irrelevant conversation time following the implementation of audio-visual recording in the surgical environment (4.2 to 1.4% of surgical time), though a link between irrelevant conversation times and adverse events was not established in this study [18]. Through an observational study, Tschan et al. found that increased irrelevant conversation time was independently correlated with an increased incidence of surgical site infections, conversely case relevant communication associated with a lower incidence, highlighting the clinical value of audio-visual data [19].

### Remote video auditing in surgery

Remote video auditing (RVA) is the recording’s subsequent critique of behaviours to promote safety and efficacy in various settings. In a general healthcare setting, Amerllino et al. assessed hand hygiene rates before and following implementation of RVA; an improvement in hygiene rates from 10 to 81.6% was witnessed amongst healthcare workers. The increased hand hygiene ratings were sustained during the 75-week feedback period; prolonged studies investigating the impact of RVA on infection rates amongst patients may justify the installation of such systems [20].

RVA in a surgical setting was conducted in 2016 to investigate compliance with the surgical safety checklist as well as case turnover times, the recording of the surgical environment and the auditing of the surgical footage with real-time feedback served to improve efficiency in scheduled surgery [21]. Feedback in this context was informed team members of the status of completion of different aspects of the checklist and patient/operating room (OR) status via a visual display board and text notifications [21].

Retrospective RVA analysis includes event transcription and analysis by a multidisciplinary team, to help identify deviations that can be addressed such as poor organisation or suboptimal dynamics [22]. Such critical findings per institution should focus on surgical practice positively.

### Video-based education and training

Systematic reviews on the effectiveness of video-based surgical education compared to conventional methods indicate the former as an effective and equivalent tool. This is based on outcome measures such as trainee satisfaction gauged by participant questionnaires and improved operative performance [23–25]. Such findings suggest a high-quality and standardised video library is valuable to current trainees, with research showing public video-sharing platforms such as YouTube to be the most popular resource for case preparation [26].

In 2018, LAP-VEGaS guidelines on reporting laparoscopic videos for education were developed to ensure resources met adequate standards due to concerns about the quality of educational resources and the lack of peer review. Guidelines were developed based on a Delphi process amongst an international cohort of surgical training experts, to facilitate the improvement of video-based education and reduce variability in academic rigour, enabling peer review on established guidelines [27, 28]. This is particularly important with the increasing use of video-based resources in the recent COVID-19 pandemic, having shown to be an effective teaching method in learning basic surgical skills [29].

In 2017, Hu et al. compared teaching in the operating room to video-based coaching, which resulted in significantly more teaching points per hour (102.7 vs. 63.0), and greater insight into the learning needs of residents [30]. Operating room teaching will have no substitute, but surgical students have found video atlases to be an adjunct to anatomical procedural steps [31].

Mendez et al. demonstrated significant benefits of video-based coaching amongst senior otolaryngology trainees performing complex neck dissection. The use of a narrated instructional video the day prior to operating led to reduced surgical error and staff takeover events [32]. For general surgery residents performing laparoscopic right colectomy, the review of an 18-min narrated instructional video compared to standard preparation was effective in promoting independence, with surgeons requiring less verbal assistance and improving surgical performance on a validated assessment scale [33]. Findings supporting video-based coaching exist across numerous surgical specialties, with more significant improvements witnessed amongst surgical residents than medical students [34].

### Teleconferencing/telemedicine

Besides recorded surgical video, video technology is often utilised as a live collaborative tool in surgery. Technological advancements in recent years have broadened opportunities for collaboration between hospitals and facilitated the sharing of specialised knowledge virtually via telemedicine. Recent literature has indicated a rapid uptake of telemedicine since the start of the COVID-19 pandemic owing to increased travel restrictions and infection control measures [35, 36].

Telemedicine is broadly defined as a subset of e-health which utilises communication networks to facilitate the delivery of medical expertise and services between remote sites [37]. It is an evolving tool, with numerous applications across multiple surgical specialties, with evidence of utilisation in the delivery of care to rural locations and zones of conflict, to disseminating surgical knowledge to resource-limited regions [38].

Amongst surgeons, telemedicine is primarily applied in the form of teleconferencing and telementoring. However, it has also been successfully applied for surgical consultations during the pandemic period being favoured by patients when travel and infection control restrictions were implemented [39]. Nevertheless, limitations in telemedicine visits suggest it cannot completely replace perioperative consultations, with surgeons unable to examine and assess health status accurately [40]. Despite limitations, research indicates significantly higher utilisation of telehealth across all surgical specialties post-pandemic compared to pre-pandemic [41].

Demartines et al. assessed the value of telemedicine as a collaborative tool for surgical education and patient care amongst six international university hospitals over 2 years. The consultations between these sites increased the rate of therapeutic advice from 55 to 95% due to interactivity between participating surgeons following case presentations. Eighty-six percent of the surgeons expressed satisfaction with the educational quality of teleconferences, which guided challenging patient cases contributing to case resolution [42].

In medical students, teleconferencing was found to enable more open communication, with students asking four times as many questions than when in the operative environment; students also had fewer unanswered questions compared to the OR [43]. Post-intervention questionnaires revealed that more students found educational value in the teleconferencing teaching sessions as opposed to in-person OR teaching [43]. Favourable results were also witnessed in a robotic tele-mentorship programme in 2021 amongst high school students participating in surgical training simulation tasks. Positive questionnaire outcomes indicated technical feasibility and increased independence [44].

A systematic review in 2018 assessing the value of telementoring in surgery suggested that educational outcomes with telementoring were equivalent/superior to in-person teaching in 67% of included studies [45]. Amidst the COVID-19 pandemic or in unplanned migration due to conflict, where a decline in medical specialists may have led to an increase in mortality rates [46], such findings are even more pivotal where novel solutions to deliver surgical care safely are paramount [47].

The opportunity for innovative remote hands-on surgical skill courses using telemedicine fulfils educational goals. In 2020, a hands-on hernia course was successfully conducted in this manner—the success in transitioning from an in-person course to a remote course demonstrates the ability of telementoring to bolster surgical training [48].

### Video labelling

In 2012, 312.9 million surgeries occurred with associated expected complication rates [49]. Laparoscopy and robotics inherently facilitate the recording of surgical video; however, the surgical environment external to the endoscope is not included routinely. In South Korea, a response to surgical malpractice was to mandate recording the entire OR [50].

Beyond the use of surgical video for apparent extractable metrics and live exchanges of knowledge is the ability for deeper analysis through AI and machine learning applications. Video labelling assigns meaningful information to different aspects of unedited video, including visual and temporal features. Through annotating video datasets and training AI, studies have demonstrated degrees of success in the automatic segmentation of surgical video into constituent steps in laparoscopic cholecystectomy and sigmoidectomy [51–53]. This includes the identification of surgical instruments and vital anatomical structures [54].

Work in this field enables the automated indexing of surgical video libraries, requiring minimal human intervention. Processing can also include the removal of irrelevant or patient-identifiable segments of surgical video to optimise storage and maintain patient confidentiality [55]. The application of surgical video labelling algorithms is directed towards higher reasoning functions, such as the automated calculation of operative skill metrics and intraoperative clinical decision support, providing guidance based on AI [56].

The legal frame of recording and storage are major considerations for video labelling. The potential utility of video labelling and recording in the healthcare context is promising, though lacking clarity and clear guidelines surrounding the use of video data in the healthcare context are limiting factors [57].

### Automatic video analysis

Using labelled video and AI enables practical applications in day-to-day practice, which can standardise aspects of surgical assessment. Research conducted by Khalid et al. raised the issue of impracticality of reviewing all surgical videos due to the high caseload being performed daily, also highlighting the subjectivity of individual surgeon review. The study assessed the feasibility of deep learning models to classify surgical actions (e.g. knot tying, needle passing and suturing) and estimate technical skill scores from surgical video. Its results revealed a mean precision of 91% in detecting surgical actions and a mean precision of 77% in predicting the surgical skill level of operators providing performance data which can improve patient safety [58].

A 2021 study assessing the feasibility of training an algorithm to automate the assessment of surgical skill in laparoscopic cholecystectomy demonstrated accuracy of 87 ± 0.2% for distinguishing good versus poor surgical skill [59]. Numerous studies have assessed automated methods of assessing surgical skill based on AI via kinetics such as tool, hand, eye motion tracking, analysis of muscle contractions and computer vision [60]. Applications of such automated surgical assessments range from basic to advanced surgical education, along with progression assessment for surgical licensing examinations. This may enable more objective methods of surgical assessment superior to the current premise of higher caseloads being equivalent to higher proficiency [60, 61].

As mentioned, the correlation between technical surgical skill and patient outcomes is well-established in the literature [62]. Research conducted by Hung et al. investigated the use of AI in automated surgical assessment, collecting performance metrics (APMs) via a novel Da Vinci system recording device (dVLogger). This group successfully trained a machine learning algorithm to correlate APMs with patient outcomes and predict impactful metrics such as post-operative length of stay with 88.5% accuracy, and urinary continence after prostate surgery [63, 64]. Operative parameters extracted digitally show applicability for organisations that offer open access to patient outcome data, such as the Royal College of Surgeons of England [65].

### Context-aware operating room

The unification of various data sources in the OR with technologies described above can serve towards developing algorithms with context awareness of the surgical environment.

Algorithms have shown accurate recognition of the stage of a procedure from external footage via synchronised cameras in the OR, with 84.4% accuracy in laparoscopic cholecystectomy [56]. In 2019, Bodenstedt et al. combined both visual data from endoscopic video and surgical device data and were able to train an algorithm to predict the remaining operative time in a range of laparoscopic surgical procedures [66]. Other aspects of the context-aware OR are being investigated, such as tool recognition that was successfully applied to videos of the laparoscopic cholecystectomy procedure. Through deep learning, surgical tools were classified with an average precision of 93.75% [67]. Through computer vision, human actions are also recognised through the localisation of body parts in real time, enabling the interpretation of team interactions. Last is the monitoring of radiation exposure during radiographic-guided procedures using data from the X-ray device and camera system [68].

Such work was done with the intent of improving awareness of activities occurring in the surgical environment and serving to enhance surgical workflows for all members of the team. Applications including predicting remaining time in any given procedure, automating surgical reporting and mitigating the risk of surgical errors with intraoperative decision support are likely to develop significantly over the next decade [56, 69, 70].

### Surgical data science and AI

Surgical data science refers to utilising data to extract valuable metrics and processing them to enhance an aspect of surgical practice. Maier-Hein et al. defined surgical data science, in part as “an emerging scientific field with the objective of improving the quality of interventional healthcare and its value through capturing, organization, analysis, and modelling of data” [71].

In other healthcare domains, data science has led to significant benefits being successfully applied in the fields of medical imaging and mental health [72, 73]. In the surgical field, the high-stake nature of the operating environment and the invasive nature of surgery mean adoption of such technologies is inherently more challenging.

The previous sections within this review discussed the numerous applications of video technology in surgery to date and the wealth of data contained in a surgical video recording. Video data is typically processed using convolutional neural networks (CNNs), a form of AI used primarily in the analysis of visual data [74]. The practice of surgery, as of current and historically, has relied on the judgement and surgical skill of the individual surgeon performing the procedure, though, with emerging technologies to date, this may change drastically.

If surgical data can be extracted from not only a single operating room but numerous operating rooms worldwide, multifaceted improvements may be witnessed in surgical workflow, performance, education and inevitably patient outcomes and hospital profitability [75, 76]. The future directions would encompass creating systems which have compiled and “learnt” from thousands of patients and operating rooms worldwide to provide every surgeon with intraoperative support equivalent and theoretically superior to the most experienced and technically talented surgeons [77].

The recording of the surgical field and the operative environment is increasingly common, with the increase in minimally invasive and robotic surgery necessitating an endoscope. AI through data science has shown to be able to develop the ability to distinguish and diagnose pathology from medical imaging [78], to recognise anatomical structures from operative footage [79] and to replicate and automate the performance of surgical tasks [80]. Regarding the surgical workflow, benefits would be seen with hospital schedules with the ability to predict surgical phases in real time, giving surgical staff a greater awareness of remaining procedural time [81]. Surgical data science will enable the extraction of all information relating to the surgical process and link this with patient outcomes, leading to more consistently optimal practices by the surgeons.

## Discussion and conclusion

Innovation in surgical practices has led to the speciality’s advancement and the optimisation of patient care. Recent technological innovation offers the potential to identify factors conducive to optimal surgical practice, evolving surgical education and identifying the stance of data science in the field. The preliminary and early clinical research highlights the potential utility of surgical technology, artificial intelligence and the research applications which exist when data is collected from the OR, particularly operative video.

However, the progression of this field is closely related to the availability of high-quality data libraries, in this case, surgical video. There exist publicly available datasets for use by research societies, such as the Cholec80 dataset [52], though these are limited. A systematic review of machine learning models makes evident that to curate algorithms for labelling and processing more complex procedures, more extensive datasets are required; however, improving computing power and algorithms may facilitate this [82]. Nonetheless, the requirement for manual annotation and processing remains a barrier to the development of computer vision. A scalable solution to this would be crowdsourcing to non-specialist personnel though this cannot be done for more technical steps whereby recruitment of surgical personnel would be necessary [83].

Compiling sufficient volumes of surgical data can be problematic in the standard operating room, whereby infrastructure for collecting and sharing data still needs to be implemented. Nevertheless, Maier-Hein et al. have detailed an extensive roadmap identifying many barriers and their solutions to translate proven clinical concepts into surgical practice, making evident that the evolution of surgical practice will require significant cultural shifts amongst surgical societies and patient populations to increase awareness and acceptability of digital solutions [83]. Moreover, legal and ethical considerations also remain a barrier regarding the collection and use of patient data.

Translating novel solutions to clinical practice will require mass interdisciplinary collaboration, establishing an agreed roadmap to integrate data collection, processing and careful integration and trialling of digital solutions. Efforts should be made to increase acceptability of video recording in the OR by educating relevant parties on the future benefits and facilitating partnerships between surgical centres and commercial infrastructure providers. Furthermore, it is pertinent to continue establishing and growing collaborative networks of data scientists and surgical researchers to increase awareness of the benefits of sharing data with the development of standardised protocols and confidentiality measures. Surgeons should be mindful of ethical and legal procedures surrounding the utilisation of patient data and comprehensive guidelines should be established to ensure the protection of patient confidentiality.

The OR has evolved throughout the decades. With the advent of smart technology, mass data storage and connectivity should be installed and capitalised globally to benefit health providers and patients.

## Data Availability

All data generated or analysed during this study are included in this published article and the reference list.
